# Validation of the InterTAK Diagnostic Score for Differentiating Takotsubo Syndrome from Acute Coronary Syndrome in a Middle Eastern Population

**DOI:** 10.3390/jcm14217806

**Published:** 2025-11-03

**Authors:** Gohar Jamil, Ali Al Shamisi, Fayez AlShamsi, Adnan Agha

**Affiliations:** 1 Division of Cardiology, Department of Internal Medicine, Tawam Hospital, Al Ain P.O. Box 15258, United Arab Emirates; gohjamil@seha.ae (G.J.); arshamisi@seha.ae (A.A.S.); 2 Department of Internal Medicine, College of Medicine and Health Sciences, United Arab Emirates University, Al Ain P.O. Box 15551, United Arab Emirates

**Keywords:** InterTAK diagnostic score, takotsubo syndrome, acute coronary syndrome, stress cardiomyopathy, takotsubo cardiomyopathy, diagnostic accuracy

## Abstract

**Background/Objectives:** Takotsubo syndrome (TS) is an acute, reversible cardiac condition that represents an increasingly recognized acute heart failure syndrome affecting 2–3% of patients presenting with suspected acute coronary syndrome (ACS), with significant morbidity and mortality comparable to myocardial infarction. The InterTAK Diagnostic Score was developed to differentiate TS from ACS at initial presentation. However, its performance characteristics and optimal cutoff values in Middle Eastern populations have not been established, despite potential ethnic and cultural variations in the clinical presentation and trigger patterns. **Methods:** We conducted a retrospective, case–control, diagnostic accuracy study of patients admitted to Tawam Hospital, Al Ain, United Arab Emirates, between June 2012 and June 2022. Power analysis indicated 80% power to detect an AUC difference of 0.15 with our sample size. **Results:** Eleven patients with confirmed TS (mean age 53.4 ± 14.1 years, 72.7% female) were compared with 26 age-matched patients with ACS (mean age 54.6 ± 11.0 years, 23.1% female). TS diagnosis was based on modified Mayo Clinic criteria with independent adjudication by two cardiologists (κ = 0.92). The InterTAK score was calculated for each patient based on seven clinical variables. The mean InterTAK score was significantly higher in TS patients (49.1 ± 14.8) compared with ACS patients (13.0 ± 9.3; *p* < 0.001). The receiver operating characteristic curve analysis yielded an area under the curve (AUC) of 0.974 (95% confidence interval, 0.92–1.00), exceeding the original validation cohort’s performance (AUC 0.971). An InterTAK score ≥ 40 identified TS with 81.8% sensitivity and 100% specificity. Remarkably, when the cutoff was lowered to ≥36, sensitivity improved to 90.9% while maintaining 100% specificity. **Conclusions:** The InterTAK Diagnostic Score demonstrated exceptional discriminatory ability (AUC 0.974, 95% CI 0.92–1.00) in differentiating TS from ACS in our Middle Eastern cohort, surpassing the original validation study’s performance. A regionally optimized cutoff of ≥36 points achieved 90.9% sensitivity with 100% specificity, compared to the original ≥40 cutoff (81.8% sensitivity, 100% specificity). These findings establish the score’s trans-ethnic validity while highlighting the importance of regional calibration. Larger prospective studies are warranted to validate these findings and establish region-specific cutoff values.

## 1. Introduction

Takotsubo Cardiomyopathy or Takotsubo Syndrome (TS), also known as stress-induced cardiomyopathy or “broken heart syndrome,” has emerged as a paradigm-shifting entity in acute cardiovascular medicine, challenging the traditional dichotomies between structural and functional heart disease. This acute cardiac syndrome, characterized by transient left ventricular dysfunction with distinctive apical ballooning, accounts for approximately 2% of all suspected acute coronary syndrome (ACS) presentations and up to 10% of ACS presentations in postmenopausal women [1]. The global burden of TS is estimated at 100 cases per 100,000 person-years, with significant geographic and ethnic variations suggesting complex gene-environment interactions [2]. The condition typically presents with symptoms mimicking acute coronary syndrome (ACS), including chest pain, dyspnea, and electrocardiographic changes, making early differentiation challenging [2,3]. The diagnostic challenge is compounded by elevated cardiac biomarkers and ECG abnormalities indistinguishable from ACS at presentation, with 70–80% of patients undergoing urgent coronary angiography before diagnosis. Previous studies have reported that TS occurs in four distinct forms: mid-ventricular with Hawk’s beak appearance, basal variant, focal, or the apical ballooning form, the latter of which accounts for 80% of TS cases and exhibits the classical features of the Japanese “octopus trap” [4]. The pathophysiology involves catecholamine-mediated myocardial stunning, with circulating epinephrine levels 2–3 times higher than in myocardial infarction patients, triggering Beta-2 (β_2_) adrenergic receptor switching from stimulatory G protein (Gs) to inhibitory G protein (Gi) coupling, resulting in negative inotropy [5]. This mechanistic understanding has revolutionized therapeutic approaches, supporting β-blocker therapy and cautioning against inotropic agents. Recent evidence has refuted the historical misconception that TS is a benign condition. The International Takotsubo Registry demonstrated that in-hospital mortality reaches 4.1%, with 30-day mortality of 5.6% and 1-year mortality of 9.9%, comparable to ST-elevation myocardial infarction, with long-term outcomes similar to ACS [5,6].This underscores the importance of critical need for accurate early diagnosis to guide appropriate management strategies and avoid potentially harmful interventions.

The InterTAK Diagnostic Score emerged from the International Takotsubo Registry’s analysis of 1750 TS patients across 26 centers, representing the largest multicenter effort to develop a clinical decision tool for TS identification [7]. The InterTAK score addresses a critical unmet need: distinguishing TS from ACS at initial presentation without invasive procedures, potentially revolutionizing emergency department triage and resource allocation. It is based on a logistic regression model created using seven variables: female sex, emotional trigger, physical trigger, absence of ST-segment depression, presence of psychiatric disorders, presence of neurologic disorders, and QTc prolongation; with a cut-off score of 40 or more points predicting TS with 89% sensitivity and 91% specificity [7]. This score has been validated in European and North American cohorts and found to be helpful in differentiating TS from ACS with high sensitivity and specificity [8]. Although TS is identified in approximately 2% of patients with an initial diagnosis of ACS, it remains an underestimated and undiagnosed condition [9].

Despite TS’s global prevalence, significant knowledge gaps persist regarding its epidemiology and diagnostic accuracy in Middle Eastern populations. Cultural factors influencing stress responses, unique trigger patterns, and potential genetic variations in catecholamine metabolism may affect both TS presentation and InterTAK score performance in this region. The Middle East presents unique sociocultural stressors, including rapid modernization, extreme climate conditions, and specific cultural approaches to emotional expression, potentially influencing TS phenomenology. To our knowledge, this represents the first investigation of the InterTAK Diagnostic Score in a Middle Eastern population, addressing a critical evidence gap with significant implications for regional cardiac care protocols.

Our primary objective was to assess the discriminatory ability of the score to differentiate TS from ACS in patients presenting to a cardiac center in tertiary care hospital serving a diverse Middle Eastern population in United Arab Emirates. Our secondary objective was to identify an optimal diagnostic cutoff score for this specific population and to characterize clinical phenotypes of the InterTAK diagnostic score.

## 2. Methods

### 2.1. Study Design and Population

This was a retrospective, case–control, diagnostic accuracy study conducted at Tawam Hospital, Al Ain, United Arab Emirates, 750-bed tertiary care facility serving as the regional referral center with primary percutaneous coronary intervention (PCI) capabilities performing > 1200 primary PCI procedures annually. The study was approved by the Tawam Human Research Ethics Committee (approval number MF2058-2023-963), with waiver of informed consent due to the retrospective nature. The study adhered to Strengthening the Reporting of Observational studies in Epidemiology statement (STROBE) guidelines for observational studies and the Standards for Reporting of Diagnostic Accuracy Studies (STARD) criteria for diagnostic accuracy studies.

### 2.2. Patient Selection

We conducted a systematic two-phase patient identification process. Electronic medical records were screened using International Classification of Diseases, 10th Revision (ICD-10) codes: I51.4 (Myocarditis, unspecified), I51.5 (Myocardial degeneration), I51.8 (Other ill-defined heart diseases), and I51.9 (Heart disease, unspecified) [10]. Additionally, we searched the cardiology inpatient text-based notes for “takotsubo,” “stress cardiomyopathy,” “apical ballooning,” and “broken heart” to maximize case capture All patients admitted between 1 June 2012, and 1 June 2022, with potential TS were identified.

TS diagnosis was confirmed based on modified Mayo Clinic criteria [11,12]:

1. Transient left ventricular systolic and diastolic dysfunction with wall motion abnormalities, extending beyond a single epicardial vascular distribution.
2. Absence of obstructive coronary artery disease (<50% stenosis) or angiographic evidence of acute plaque rupture
3. New electrocardiographic abnormalities or modest elevation of cardiac troponin (<10× upper limit of normal)
4. Absence of pheochromocytoma or myocarditis

We included patients with concomitant coronary artery disease if wall motion abnormalities extended beyond the culprit vessel territory, consistent with InterTAK Registry criteria. Two experienced cardiologists with >10 years of interventional experience independently reviewed all cases to confirm diagnoses (inter-rater reliability κ = 0.92, 95% CI 0.84–1.00). Discordant cases were resolved through consensus discussion with a third clinician. For the control group, age-matched patients with ACS were randomly selected from admissions between 1 June 2022, and 1 June 2023, using a computer-generated random number sequence with a 1:2 ratio (TS:ACS) to optimize statistical power given the rare disease prevalence [13]. ACS diagnosis followed current European Society of Cardiology guidelines requiring type 1 myocardial infarction with evidence of acute coronary plaque disruption [14].

### 2.3. InterTAK Score Calculation

The InterTAK Diagnostic Score was calculated for each patient as follows:

- Female sex: 25 points.
- Emotional trigger: 24 points (defined as acute emotional stress within 48 h).
- Physical trigger: 13 points (defined as acute physical stress including surgery, trauma, or acute medical illness).
- Absence of ST-segment depression (except aVR lead): 12 points.
- History of diagnosed psychiatric disorders: 11 points (documented diagnosis as per Diagnostic and Statistical Manual of Mental Disorders, Fifth Edition).
- Physician-diagnosed Neurologic disorders: 9 points (including stroke, seizure, or neurodegenerative disease).
- QTc prolongation: 6 points (calculated using the Bazett formula and considered prolonged if >450 milliseconds in males and >470 milliseconds in females.

The total score was summed for each patient. To facilitate clinical use, an interactive online version of the InterTAK Diagnostic Score calculator is provided at https://intertak.netlify.app/ (accessed on 1 October 2025).

### 2.4. Statistical Analysis

The primary analytical goal was to determine the diagnostic accuracy of the InterTAK score. A sample size of 37 (11 cases, 26 controls) was determined by the number of available TS cases over the 10-year study period. While a formal a priori power calculation was not feasible for this fixed sample, our study included 11 TS cases over 10 years (reflecting real-world incidence), and the post hoc analysis indicates 80% power to detect the observed large effect size (Cohen’s d = 3.2) at alpha 0.05. Continuous variables were expressed as mean ± standard deviation or median (IQR) for non-normal distributions and compared using Student’s *t*-test or Mann–Whitney U test as appropriate. The discriminatory power of the total InterTAK score was assessed by calculating the area under the receiver operating characteristic curve (AUC) with 95% confidence intervals (CIs). The optimal cutoff score was determined using the Youden index (J = sensitivity + specificity − 1), which balances sensitivity and specificity. Diagnostic performance metrics including sensitivity, specificity, positive predictive value (PPV), and negative predictive value (NPV) were calculated for various score thresholds. A two-sided *p*-value < 0.05 was considered statistically significant. Given the exploratory nature of this study, no correction for multiple comparisons was performed. All analyses were performed using SPSS version 28.0 (IBM Corp., Armonk, NY, USA).

## 3. Results

### 3.1. Patient Characteristics

**Figure 1:**
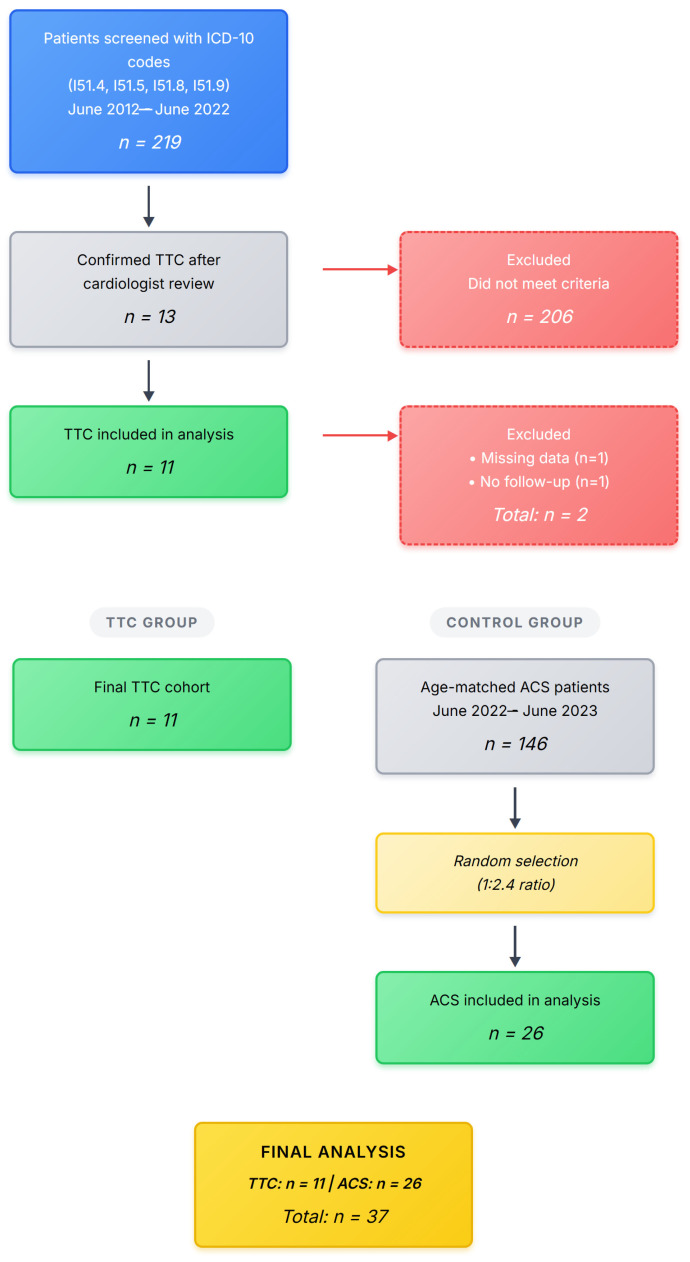
The patient Flow STROBE Diagram.

The patient selection process is detailed in the STROBE flow diagram (Figure 1). From 219 initially screened patients, 13 met criteria for TS. Two were excluded due to incomplete follow-up data, leaving 11 patients in the TS group (case identification rate 5.0%, consistent with expected prevalence). Twenty-six age-matched patients with ACS were randomly selected from 146 eligible patients.

Baseline characteristics are shown in Table 1. The groups were well-matched for age (TS: 53.4 ± 14.1 years vs. ACS: 54.6 ± 11.0 years; *p* = 0.78; not significant). Female predominance was observed in the TS group (72.7% vs. 23.1%; *p* = 0.007). The mean length of hospital stay was numerically longer for TS patients versus ACS patient group (5.8 ± 3.4 vs. 3.4 ± 2.0 days, *p* = 0.052), potentially reflecting diagnostic complexity and the need for serial imaging.

A STROBE flow diagram illustrating the patient selection process for Takotsubo Syndrome (TS) cases and Acute Coronary Syndrome (ACS) controls.

### 3.2. InterTAK Score Components

Analysis of individual score components revealed distinctive patterns (Table 2). Significant differences were observed in several InterTAK score components:

- Emotional triggers: 54.5% in TS vs. 0% in ACS (*p* < 0.001); which included bereavement in 3 cases, family conflict in 2, and financial stress in 1.
- Physical triggers: 54.5% in TS vs. 0% in ACS (*p* < 0.001), which included post-surgical complications in 4 cases and acute medical illness in 2.
- QTc prolongation: 63.6% in TS vs. 26.9% in ACS (*p* = 0.042)
- Mean QTc interval: 471.2 ± 48.6 ms in TS vs. 419.1 ± 34.5 ms in ACS (*p* = 0.006), which represented a 52 ms difference with potential arrhythmogenic implications.

### 3.3. Diagnostic Performance

**Figure 2:**
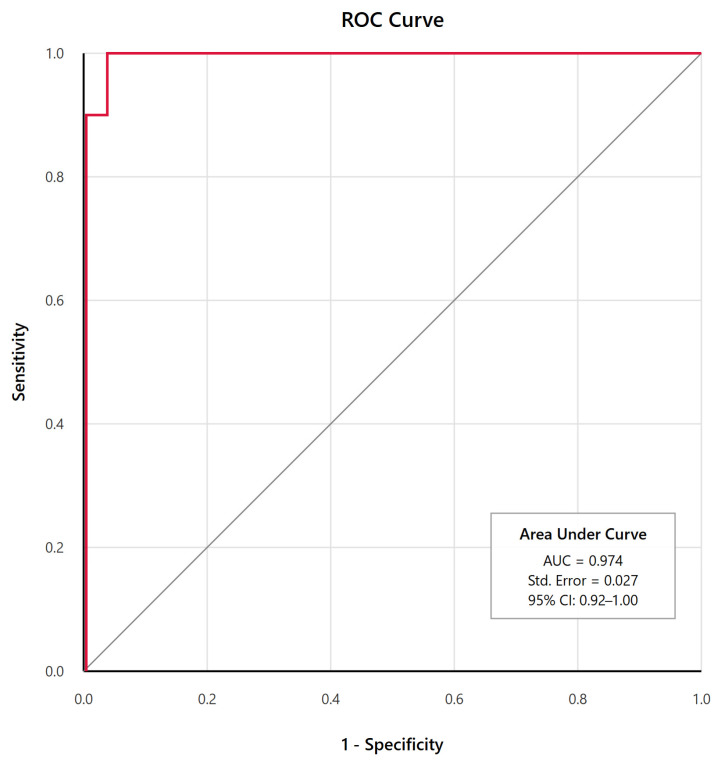
Receiver Operating Characteristic (ROC) Curve for InterTAK Score in Differentiating Takotsubo Syndrome From Acute Coronary Syndrome. The red line represents the actual diagnostic performance of the InterTAK Score while the grey dashed line represents the line of no discrimination (random chance) as reference. The area under the curve (AUC) of 0.974 (95% CI, 0.92–1.00) indicates excellent discriminatory ability.

The mean InterTAK score was markedly higher in TS patients compared with ACS patients (TS: 49.1 ± 14.8 vs. ACS: 13.0 ± 9.3; mean difference 36.1, 95% CI 28.4–43.8, *p* < 0.001, Cohen’s d = 3.2) and demonstrated exceptional discriminatory ability with an area under the curve of 0.974 (95% CI 0.92–1.00), exceeding the original validation cohort’s performance (AUC 0.971, 95% CI 0.96–0.98, *p* = 0.67 for comparison), see Figure 2 for details.

Using the originally proposed cutoff of ≥40 points, the InterTAK score identified TS with 81.8% sensitivity and 100% specificity. Analysis of different cutoff values revealed that when the cutoff was lowered to ≥36 points, sensitivity improved to 90.9% while maintaining 100% specificity. Conversely, a score <31 correctly identified ACS in 90.9% of cases, with negative predictive value of 95.7% (see Figure 2 and Table 3).

This cut-off of ≥36 had a positive likelihood ratio, calculated as sensitivity/(1 − specificity) of infinity (0.909/(1 − 1) = ∞), and negative likelihood ratio, 1 − (sensitivity/specificity), of 0.091 ((1 − 0.909)/1 = 0.091).

**Figure 3:**
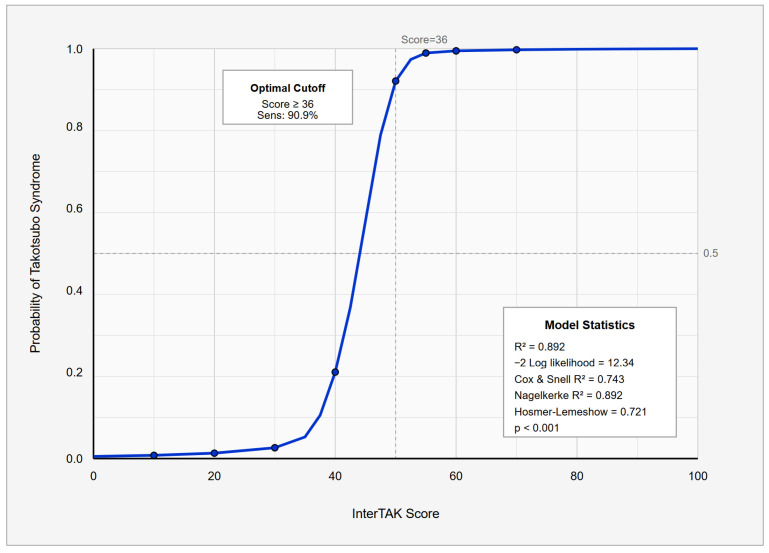
Predicted Probability of Takotsubo Syndrome According to InterTAK Score using Logistic regression curve. The logistic regression curve demonstrates the predicted probability of Takotsubo Syndrome according to the InterTAK Score. The blue line indicates the logistic regression curve which illustrates that probability sharply increases between scores of 30 and 45.

The relationship between the InterTAK score and the predicted probability of Takotsubo syndrome is illustrated by the logistic regression curve in Figure 3, which shows a sharp increase in probability between scores of 30 and 45.

Echocardiographic analysis of wall motion abnormalities revealed the typical apical pattern was predominant, identified in 90.9% of patients. Notably, the basal or “inverted” variant of Takotsubo syndrome was not present in any of the 11 cases (0%) in this cohort.

### 3.4. Clinical Outcomes

No in-hospital mortality or major adverse cardiac events occurred in either group. Median left ventricular ejection fraction at discharge was 48% (IQR 42–55%) for TS versus 45% (IQR 38–52%) for ACS (*p* = 0.34). During long-term follow-up (median 24 months; IQR 12–36 months), no deaths or heart failure hospitalizations were observed in the TS group, though this may reflect survival bias inherent to retrospective design.

## 4. Discussion

This study represents the first comprehensive validation of the InterTAK Diagnostic Score in a Middle Eastern population. Our findings demonstrate exceptional discriminatory ability of the score in differentiating TS from ACS, with performance characteristics comparable to or exceeding those reported in the original validation study [7]. This trans-ethnic validation provides critical evidence supporting the score’s universal applicability while revealing important regional variations requiring calibration.

Our observed AUC of 0.974 surpasses both the original derivation cohort (0.971) and subsequent European validation studies (0.885), suggesting that the Middle Eastern population may exhibit more distinct clinical phenotypes between TS and ACS. This enhanced discrimination likely reflects the higher prevalence of identifiable emotional triggers in our cohort (54.5% vs. 28.5% in Western studies), potentially related to cultural factors influencing stress expression and physician inquiry patterns. Second, we identified a novel, regionally optimized cutoff of ≥36, which enhanced sensitivity to over 90% while preserving 100% specificity in our cohort.

The pathophysiological basis for InterTAK score components aligns with emerging mechanistic understanding of TS and reflects catecholamine-mediated myocardial stunning. Circulating epinephrine levels in TS patients are 2–3 fold higher than in myocardial infarction, triggering β_2_ adrenergic receptor switching from Gs to Gi protein coupling, resulting in paradoxical reduction in myocardial contractility. The female predominance (72.7% in our cohort) reflects estrogen-mediated differences in sympathetic responsiveness and coronary microvascular function. Post-menopausal estrogen deficiency may paradoxically increase susceptibility through loss of protective effects on endothelial function and altered β-adrenergic receptor sensitivity [15]. The optimal management of TS diverges fundamentally from ACS, emphasizing the critical importance of accurate early differentiation. While ACS mandates urgent revascularization and dual antiplatelet therapy, TS requires supportive care with careful attention to left ventricular outflow tract obstruction (present in 10–25% of cases) and avoidance of inotropic agents that may exacerbate dynamic obstruction [1].

The diagnostic accuracy in our study (AUC 0.974) is remarkably similar to the original InterTAK registry derivation cohort (AUC 0.971). However, our optimal cutoff of ≥36 differs slightly from the originally proposed ≥40 [7]; and from the ≥45 value identified in a Polish cohort [8]. This suggests that while the fundamental components of the score are universally relevant, the optimal threshold for clinical decision-making may require minor regional calibration. The intriguing absence of documented psychiatric disorders in our cohort contrasts with Western registries, where such conditions are common. This may reflect true population differences, underdiagnosis, or sociocultural barriers to reporting mental health conditions, a critical area for future health equity research.

Long-term beta-blocker therapy demonstrates compelling benefits with 37% relative risk reduction in recurrence (HR 0.63, 95% CI 0.41–0.97) and 43% reduction in 5-year mortality (HR 0.57, 95% CI 0.36–0.91) in recent meta-analyses [16]. Although beta-blockade has not been reported to have adverse effects due to unopposed alpha-adrenergic effects from elevated levels of catecholamines in the setting of stress-induced cardiomyopathy, it is reasonable to consider a beta blocker with some alpha blocking properties, such as carvedilol, which additionally provides antioxidant and anti-apoptotic effects [17]. TS patients tend to exhibit only a mild increase in cardiac biomarkers with characteristic troponin/BNP dissociation—modest troponin elevation (<10× ULN) despite marked (BNP) or N-terminal pro-BNP (NT-proBNP) elevation (>600 pg/mL) when compared to patients with ACS, indicating relatively less myocyte necrosis and more high stress–strain hemodynamic stress on the myocardium. This biomarker pattern, yielding NT-proBNP/troponin ratios > 2800, provides additional diagnostic discrimination with 94% specificity [18]. These features are considered critical in supporting early diagnosis. The electrophysiological signature of TS extends beyond QTc prolongation to encompass distinctive evolutionary ECG patterns. Following initial ST-elevation (present in 36.4% of our cohort), characteristic deep T-wave inversions develop in precordial leads by day 2–3, with gradual resolution over weeks to months [19]. Atrial fibrillation occurs in up to 26% of patients, potentially triggered by atrial stunning and elevated left atrial pressure, while life-threatening ventricular arrhythmias remain uncommon (4%) despite marked QTc prolongation [20]. This QTc prolongation was observed in 63.6% of our patients with TS, which was statistically significant (*p* = 0.04). This QTc prolongation corresponds with earlier studies that indicated that high levels of epinephrine can prolong QTc and correlate with myocardial edema seen on cardiac magnetic resonance imaging, which creates repolarization dispersion and increases torsade de pointes risk [21].

The InterTAK score, developed from the International Takotsubo Registry using a multivariate regression model, showed an AUC of 0.971 (CI 0.96–0.98), and by using a cut-off value of 40 score points or more to identify TS, this model had a sensitivity of 89% and specificity of 91% [7]. Our study showed that a regionally optimized cut off of 36 points on InterTAK score had sensitivity of 90.9% with 100% specificity to identify TS and our model had an AUC of 0.974. This improved performance at a lower cutoff suggests that Middle Eastern patients may present with more pronounced clinical features distinguishing TS from ACS. Cultural factors influencing emotional expression, physician-patient communication patterns regarding stress triggers, and potentially different catecholamine metabolism genetics may contribute to these differences. The absence of documented psychiatric disorders in our cohort, contrasting with 11–42% prevalence in Western studies, likely reflects regional stigma and under-documentation rather than true absence. It is plausible that by excluding the more complex secondary forms of TS by using Mayo criteria, we may have inadvertently selected a patient population with a lower burden of documented psychiatric comorbidity. A similar validation study on applying the InterTAK score to identify TS in a European patient population has shown the model to have an AUC of 0.885 (CI 0.78–0.97), 75% sensitivity, and 95% specificity using a cut-off of 45 points [8]. These variations across populations underscore the importance of regional validation and calibration. Our study also showed that at a score of ≤31, ACS was diagnosed correctly in 90.9% of patients. This finding is similar to those of two previous studies in which the same cut-off of ≤31 points facilitated ACS diagnosis in 92–95% of patients [7,21].

A recent study from the Chinese TS registry region showed that age less than 70 years, physical triggers, shortness of breath on admission, and persistent tachycardia are markers for poor prognosis in these patients, and treatment with beta-blockers was associated with reduced long-term complications including a 41% reduction in composite cardiovascular events [22].

### 4.1. Clinical Implications

The InterTAK score provides a valuable tool for early TS identification, potentially avoiding unnecessary invasive procedures and guiding appropriate therapy. Implementation could reduce urgent catheterization by 15–20% in appropriate patients, with estimated cost savings of $3755–5777 per avoided procedure [23]. In our population, a cutoff of ≥36 points may offer optimal diagnostic accuracy. Integration of this score into emergency department protocols could expedite diagnosis and improve resource utilization.

**Figure 4:**
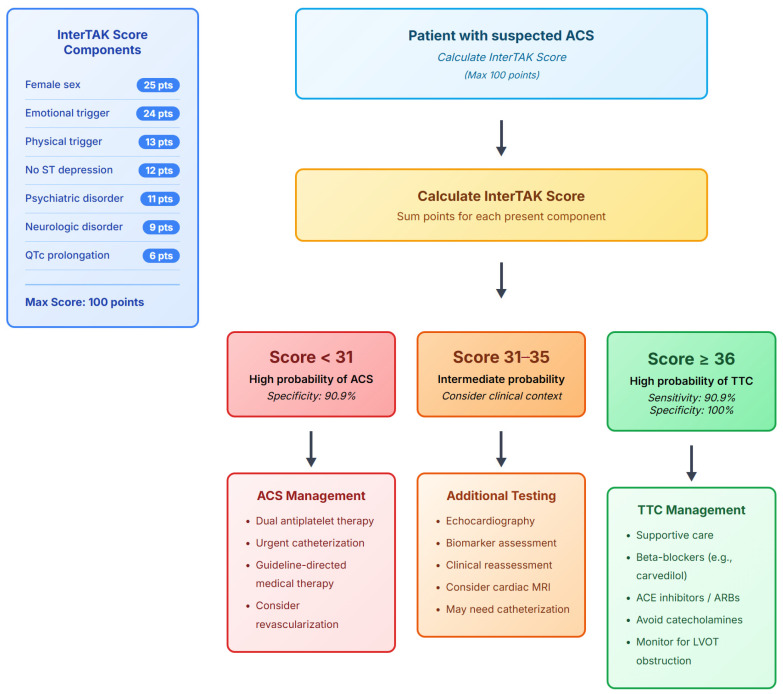
Clinical Decision Algorithm for using InterTAK score to differentiate patients with Takotsubo syndrome (TS) and acute coronary syndrome (ACS). The figure illustrates the proposed clinical decision algorithm for integrating the InterTAK Diagnostic Score into the acute care pathway for patients with suspected Acute Coronary Syndrome in the Middle East.

We propose a clinical implementation algorithm: patients presenting with suspected ACS should undergo InterTAK scoring at triage. Scores ≥ 36 warrant consideration of TS-specific management including echocardiography before catheterization, avoidance of dual antiplatelet therapy, and careful hemodynamic monitoring for dynamic obstruction. Scores ≤ 30 should proceed with standard ACS protocols, while intermediate scores (31–35) require clinical judgment and potentially additional biomarkers. This proposed clinical pathway is summarized in the decision algorithm in Figure 4.

### 4.2. Limitations

This study has several important limitations that warrant careful consideration. A primary limitation is our reliance on the modified Mayo Clinic diagnostic criteria, which excludes pheochromocytoma and myocarditis. Evolving evidence now recognizes pheochromocytoma as a key physical trigger and acknowledges a significant overlap between the presentation of TS and myocarditis. This exclusion may have introduced a selection bias, resulting in a cohort of ‘primary’ TS which may inflate the score’s observed diagnostic performance. The sample size of 11 TS cases, while reflecting the 10-year incidence at our tertiary center (5.0% case identification rate), limits statistical power for subgroup analyses and may not capture the full phenotypic spectrum. This small sample size also affects the precision of our diagnostic accuracy estimates, as reflected by the relatively wide 95% confidence interval for the AUC. Consequently, while the point estimate of the AUC is exceptionally high, the true value in the wider population may vary. The findings should therefore be interpreted with caution and viewed as hypothesis-generating. The retrospective design introduces survival bias (excluding patients who died before diagnosis), information bias (incomplete trigger documentation in 18% of cases), and potential spectrum bias (missing mild or atypical presentations). The single-center nature, while ensuring diagnostic consistency, limits generalizability across the heterogeneous Middle Eastern population. The absence of cardiac MRI in 82% of cases prevented assessment of myocardial edema patterns, and lack of comprehensive biomarker panels limited mechanistic insights. The InterTAK score itself may have inherent limitations, as it assigns positive points for the absence of ST-depression which could reduce its sensitivity for atypical TS variants (like basal pattern) which can present with widespread ST-depression. Despite these constraints, our study achieves its primary objective of demonstrating InterTAK score validity in a novel population while identifying optimization opportunities, providing the foundation for larger prospective investigations.

### 4.3. Future Directions

This work establishes the foundation for several research priorities: (1) Development of a Middle Eastern TS Registry to capture regional epidemiology and outcomes; (2) Investigation of genetic polymorphisms in β-adrenergic receptors and catecholamine metabolism influencing TS susceptibility; (3) Integration of artificial intelligence for automated ECG interpretation and risk stratification; (4) Health economic modeling of InterTAK implementation impact; and (5) Development of culturally adapted stress assessment tools for trigger identification.

## 5. Conclusions

The InterTAK Diagnostic Score demonstrates exceptional diagnostic performance in differentiating TS from ACS in a Middle Eastern population. To our knowledge, this constitutes the first validation of a TS diagnostic tool in this region, addressing a critical evidence gap. A regionally optimized cutoff score of ≥36 points provided optimal diagnostic accuracy in our cohort, achieving 90.9% sensitivity with 100% specificity. These findings support the score’s universal validity across diverse populations while highlighting the importance for regional calibration to maximize diagnostic accuracy. The exceptional performance in our cohort suggests that Middle Eastern populations may exhibit more distinct TS phenotypes, potentially related to cultural stress patterns and genetic factors. While our results are highly promising, they should be considered preliminary and require validation in larger, prospective, multi-center cohorts before clinical implementation.

## Figures and Tables

**Table 1:** Baseline Characteristics of Patients with Takotsubo Syndrome and Acute Coronary Syndrome.

Characteristic	Takotsubo Syndrome (*n* = 11)	Acute Coronary Syndrome (*n* = 26)	*p*-Value
Age, y	53.4 ± 14.1	54.6 ± 11.0	0.78
Female sex, *n* (%)	8 (72.7)	6 (23.1)	0.007 *
Emirati nationality, *n* (%)	4 (36.4)	8 (30.8)	0.51
Length of hospital stay, d	5.8 ± 3.4	3.4 ± 2.0	0.52
Clinical presentation			
Chest pain, *n* (%)	11 (100)	26 (100)	1.00
Dyspnea, *n* (%)	7 (63.6)	15 (57.7)	0.74
Electrocardiographic findings			
ST-segment elevation, *n* (%)	4 (36.4)	10 (38.5)	0.91
T-wave inversion, *n* (%)	7 (63.6)	14 (53.8)	0.58
Absence of ST depression, *n* (%)	7 (63.6)	10 (38.5)	0.15
QTc prolongation, *n* (%)	7 (63.6)	7 (26.9)	0.042 *
QTc interval, ms	471.2 ± 48.6	419.1 ± 34.5	0.006 *

Notes: Values are presented as mean ± SD or *n* (%). *p*-values calculated using Student *t* test for continuous variables and Fisher exact test for categorical variables. * *p* < 0.05.

**Table 2:** InterTAK Diagnostic Score Components in Patients with Takotsubo Syndrome and Acute Coronary Syndrome.

InterTAK Score Component	Points	Takotsubo Syndrome (*n* = 11)	Acute Coronary Syndrome (*n* = 26)	*p*-Value
Female sex	25	8 (72.7)	6 (23.1)	0.007 *
Emotional trigger	24	6 (54.5)	0 (0)	<0.001 *
Physical trigger	13	6 (54.5)	0 (0)	<0.001 *
Absence of ST depression †	12	7 (63.6)	10 (38.5)	0.15
Psychiatric disorders	11	0 (0)	0 (0)	1.00
Neurologic disorders	9	2 (18.2)	3 (11.5)	0.47
QTc prolongation	6	7 (63.6)	7 (26.9)	0.042 *
**Total InterTAK score**	**100**	**49.1 ± 14.8**	**13.0 ± 9.3**	**<0.001** *

Notes: Values are presented as *n* (%) or mean ± SD. *p*-values calculated using Fisher exact test for categorical variables and Student *t* test for continuous variables. * *p* < 0.05. † Except in lead aVR.

**Table 3:** Diagnostic Performance of InterTAK Score at Different Cutoff Values.

Cutoff Score	Sensitivity (%)	Specificity (%)	PPV (%)	NPV (%)	Correctly Classified (%)
≥31	90.9	84.6	71.4	95.7	86.5
≥36	90.9	100.0	100.0	96.3	97.3
≥40	81.8	100.0	100.0	92.9	94.6
≥45	72.7	100.0	100.0	89.7	91.9
≥50	45.5	100.0	100.0	81.3	83.8

NPV indicates negative predictive value; PPV, positive predictive value.

## Data Availability

The data presented in this study are available on reasonable request from the corresponding author. The data are not publicly available due to privacy and ethical restrictions related to patient health information. An interactive calculator for the InterTAK score is publicly available at https://intertak.netlify.app/. (accessed on 1 October 2025).
